# Magnetic Studies of Superconductivity in the Ga-Sn Alloy Regular Nanostructures

**DOI:** 10.3390/nano13020280

**Published:** 2023-01-09

**Authors:** Marina V. Likholetova, Elena V. Charnaya, Evgenii V. Shevchenko, Min Kai Lee, Lieh-Jeng Chang, Yurii A. Kumzerov, Aleksandr V. Fokin

**Affiliations:** 1 Physics Department, St. Petersburg State University, 198504 St. Petersburg, Russia; 2 Research Park, St. Petersburg State University, 199034 St. Petersburg, Russia; 3 Instrument Center of Ministry of Science and Technology, National Cheng Kung University, Tainan 70101, Taiwan; 4 Department of Physics, National Cheng Kung University, Tainan 70101, Taiwan; 5 Ioffe Institute RAS, 194021 St. Petersburg, Russia

**Keywords:** Ga-Sn eutectic nanoalloy, opal template, superconductivity, dc magnetization

## Abstract

For applications of nanolattices in low-temperature nanoelectronics, the inter-unit space can be filled with superconducting metallic alloys. However, superconductivity under nanoconfinement is expected to be strongly affected by size-effects and other factors. We studied the magnetic properties and structure of the Ga-Sn eutectic alloy within regular nanopores of an opal template, to understand the specifics of the alloy superconductivity. Two superconducting transitions were observed, in contrast to the bulk alloy. The transitions were ascribed to the segregates with the structures of tetragonal tin and a particular gallium polymorph. The superconducting-phase diagram was constructed, which demonstrated crossovers from the positive- to the common negative-curvature of the upper critical-field lines. Hysteresis was found between the susceptibilities obtained at cooling and warming in the applied magnetic field.

## 1. Introduction

Properties of nanolattices can be tuned by filling the voids between nano-sized units with various substances. Such a scenario opens an extensive area of new feasible applications of nanolattices in electronics and engineering. For instance, filling the inter-unit space with liquid metals or alloys could allow the production of efficient thermal-interface-materials, as was discussed recently for metallic-based composites in [1,2,3]. For applications in superconducting nanoelectronics, the inter-unit space in nanolattices can be filled with metals or alloys, which transform into superconductors at decreasing temperatures. However, superconductivity of metallic substances embedded into the inter-unit space in nanolattices can differ from that of their bulk counterparts, due to size-effects, coupling with the surface of the units and the influence of the unit’s shape, as was found in studies of nanostructured superconductors [4,5,6,7]. The regularity in the positions of the nanolattice constituent elements might also affect superconductivity in the embedded substances. The role of these factors is poorly understood, especially for metallic eutectic-alloys, whose low-temperature superconducting behavior is not well studied, even in bulk. Revealing specific features of superconductivity in eutectic alloys introduced into artificial opal matrices, which are considered as close analogs of nanolattices, can help to solve this problem.

Bulk eutectic gallium-based metallic alloys are prospective materials for applications in soft robotics, wearable electronics, and bio-devices because of stable electrical properties and non-toxicity [8,9,10,11]. In particular, the Ga-Sn alloy is employed as a superconductive solder. Since gallium alloys near the eutectic points are liquid at room temperature, they can be used as self-healing superconductors.

Being nanostructured as a result of embedding into nanoporous templates, the gallium-based alloys are expected to show remarkable changes in their superconducting features, as was found for pure gallium, binary Ga-Ag and triple Ga-In-Sn alloys [12,13,14]. In the particular case of the binary Ga-Sn alloy, superconductivity was studied only in bulk [15]. In the present article we report studies of the superconducting properties of the Ga-Sn alloy introduced into pores of an opal matrix. We measure the temperature and field dependencies of dc magnetization. We show that superconductivity for the alloy within pores appears in two steps, in contrast with the bulk alloy. The magnetic phase diagram demonstrates specific features, with crossovers between positive and negative curvatures of the upper critical-field lines.

## 2. Materials and Methods

**Figure 1:**
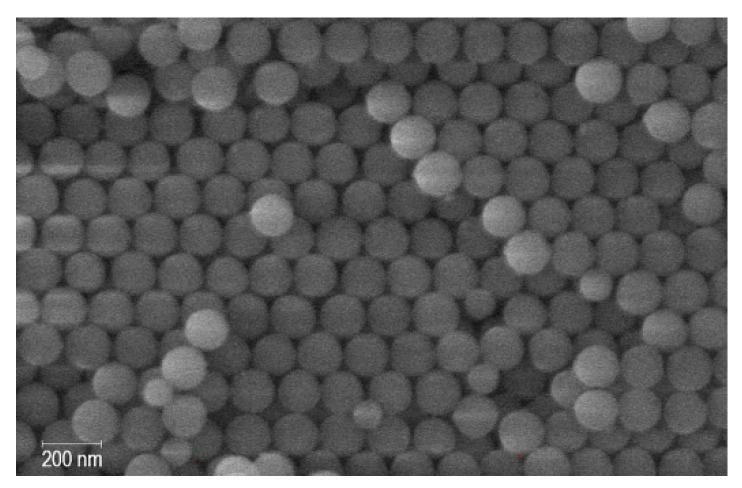
SEM image of the opal matrix.

The synthetic opal matrix is a cubic close-packing of amorphous silica spheres. The mean diameter of the silica spheres is equal to 150 nm. The SEM image is shown in Figure 1. The cubic close-packing of rigid spheres comprises a network of open octahedral and tetrahedral pores, with a total volume of 26%. The calculated sizes of the octahedral and tetrahedral pores in the ideal opal structure are 62 and 34 nm, respectively. However, sintering the packed silica balls may slightly reduce the pore dimensions.

The Ga-Sn binary eutectic-alloy has the composition 91.5 at.% Ga/8.5 at.% Sn. The alloy was introduced into the opal pores in the liquid state under a pressure of up to 20 kbar. The pore filling was near 85%, as was found by weighing the empty and loaded opal. The sample for study was cut from the loaded opal in the shape of a plate ~0.4 mm thick, with a face of approximately 4.5 × 4.5 mm. It was then cleaned of the alloy on the surface. The cleaned sample mass was 22.5 mg.

The dc magnetization, *M*, was measured using a Quantum Design MPMS SQUID magnetometer (SQUID VSM) within the range 1.8 to 10 K. The plate was oriented along the magnetic field. The temperature dependencies of *M* at various magnetic fields, *H*, up to 30 kOe were monitored upon warming, after preliminary cooling in zero field (zero-field-cooled (ZFC) protocol), upon cooling in fields (field-cooled-cooling (FCC) protocol) and upon warming in fields (field-cooled-warming (FCW) protocol). The *M(H)* hysteresis loops were measured at 2 and 5 K at fields in the range −70 to 70 kOe. The magnetic susceptibility was calculated as χ=M/H.

The X-ray powder-diffraction patterns were obtained using an Ultima IV (Rigaku) CuK_α_ diffractometer, equipped with a low-temperature chamber. Measurements were carried out at 297 and 110 K. The patterns were analyzed with PDXL (Rigaku) and Topas 5.0 (Bruker GmbH) packages and the ICDD PDF-2 2020 and ICSD 2021 databases. The image of the opal matrix surface was obtained using the Zeiss Merlin scanning electron microscope, at room temperature.

## 3. Results

**Figure 2:**
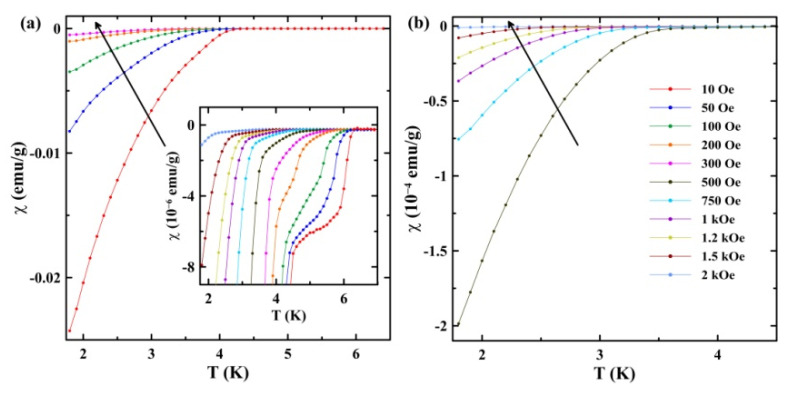
Temperature dependencies of the ZFC susceptibility at magnetic fields from 10 to 300 Oe (**a**) and from 500 Oe to 2 kOe (**b**). The arrows indicate the increase in fields. The inset shows scaled ZFC curves near the superconducting transitions.

**Figure 3:**
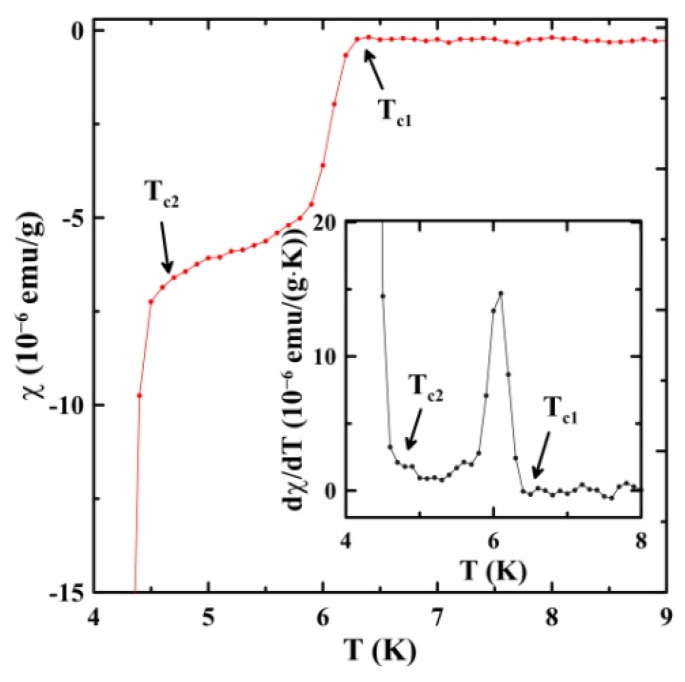
Temperature dependence of the ZFC susceptibility at 10 Oe near the superconducting transitions. The inset shows the first derivative.

Temperature dependencies of the ZFC susceptibility at fields from 10 to 300 Oe and from 500 Oe to 2 kOe are shown in Figure 2a,b, respectively. The scaled curves are presented in the inset in Figure 2a. Two superconducting transitions are seen with quite different diamagnetic screening. They continuously shift to low temperatures with an increasing magnetic bias field. Since the observed transitions are not sharp, even at low external fields, the superconducting temperatures were found as special points on the plots of the temperature dependence of the first derivative of susceptibility with temperature dχ/dT, where the derivative starts growing upon decreasing temperature. An example is shown in Figure 3. At 10 Oe, the superconducting temperatures are $T_{C1}$ = 6.4 K and $T_{C2}$ = 4.7 K.

**Figure 4:**
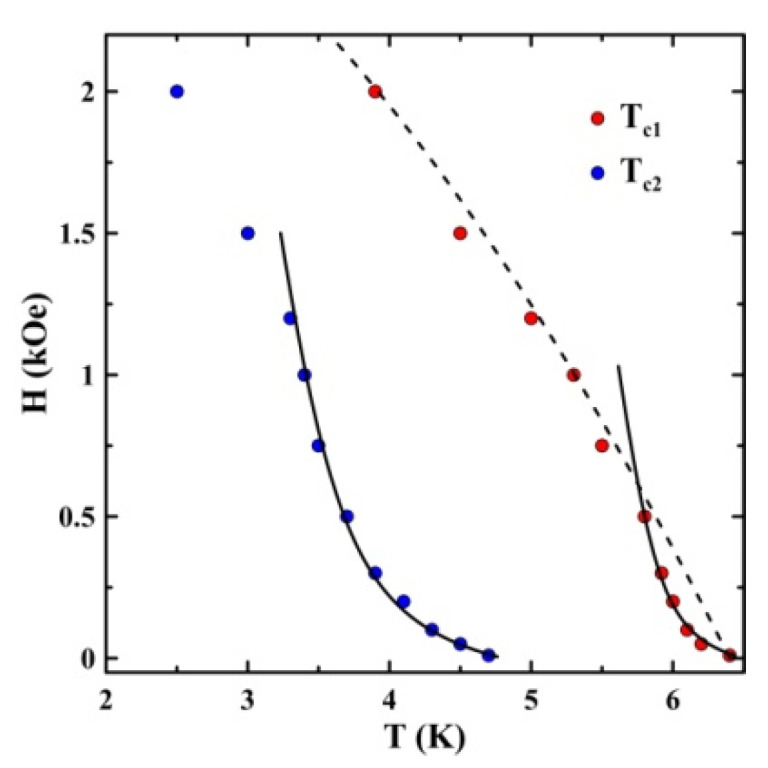
The superconducting-phase diagram obtained from magnetic measurements. The dashed line is a two-fluid model fit. Solid lines show fitting using Equation (13) from [16].

The data presented in Figure 2 were used to construct the *H*-*T* phase diagram for the superconducting transitions (Figure 4).

Figure 4 demonstrates the positive curvature for the critical lines at lower bias fields for both superconducting transitions. At stronger applied fields, the crossover to the ordinary negative curvature can be seen on both critical lines.

**Figure 5:**
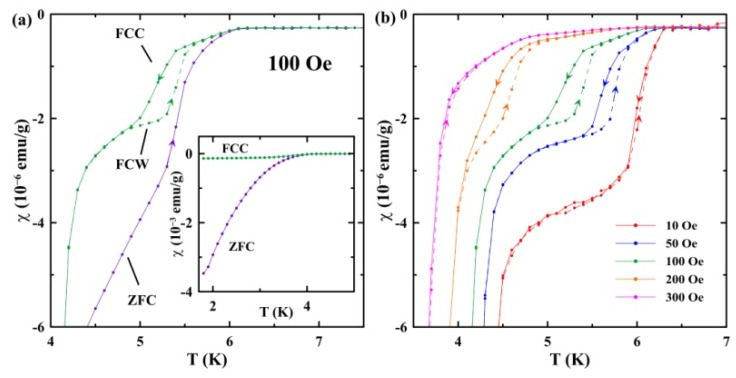
(**a**) Temperature dependencies of the FCC, FCW, and ZFC susceptibilities at 100 Oe near the superconducting transitions. The inset shows the total ZFC and FCC curves. (**b**) Temperature dependencies of the FCC (solid lines) and FCW (dashed lines) susceptibilities near the superconducting transitions at fields indicated in the panel. The arrows show the direction of changing temperature.

Figure 5a and the inset in Figure 5a show a remarkable distinction between the ZFC and FCC susceptibilities. Their ratio exceeds 30 at 2 K. However, the ZFC and FCC curves run close to each other in the temperature range of weak diamagnetism between $T_{C1}$ and $T_{C2}$. At magnetic fields from 50 to 200 Oe (Figure 5b), the FCC and FCW susceptibility curves form hysteresis loops below the first (higher-temperature) superconducting transition, the FCW susceptibility being smaller than the FCC one. Within the hysteresis loops, the FCW curves approach closer to ZFC, as can be seen for 100 Oe, in Figure 5a. Beyond the hysteresis loops, as well as at other magnetic bias fields, the FCC and FCW curves coincide.

**Figure 6:**
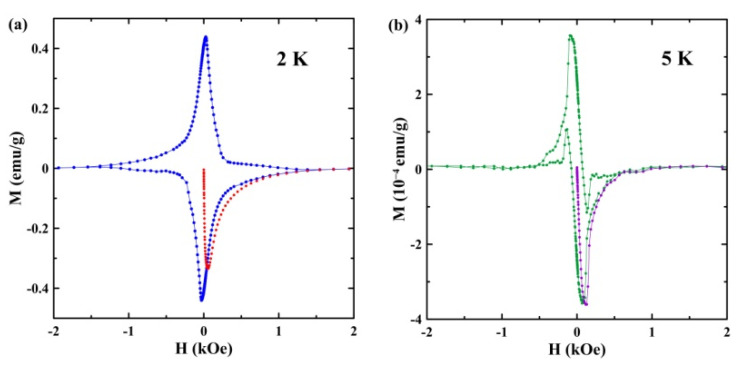
The field versus magnetization loops at 2 K (**a**) and 5 K (**b**). The red (**a**) and purple (**b**) colors show the virgin magnetization.

Figure 6 presents variations of magnetization with the magnetic field obtained at 2 K (a) and 5 K (b), which lie within the temperature ranges of strong and weak diamagnetism, respectively. The magnetization behavior at 2 K is totally irreversible, while we can see a partial reversibility at 5 K.

**Figure 7:**
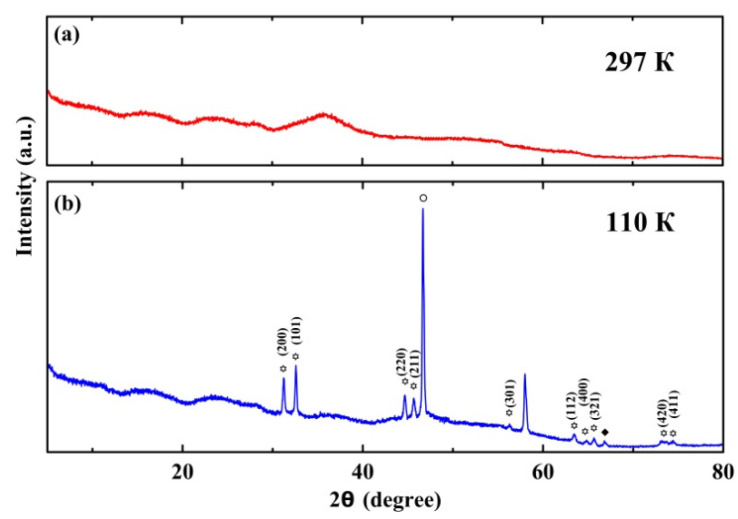
The X-ray patterns at 297 K (**a**) and 110 K (**b**). The peaks, which belong to the Sn-rich segregates, are marked with asterisks. The circle and diamond mark peaks which correspond to segregates with the structures of α-Ga and λ-Ga, respectively.

The X-ray powder patterns obtained at room temperature and at 110 K are shown in Figure 7. At room temperature there are no peaks corresponding to solid alloy within nanopores. The broad peaks emerged due to diffraction of the opal silica spheres and melted alloy. The narrow peaks in the X-ray pattern appear below 284 K upon cooling. The pattern generally does not change at further cooling down to 110 K. The attribution of these peaks will be discussed below.

## 4. Discussion

The bulk Ga-Sn alloy segregates below the solidus into the Ga-rich and Sn-rich phases with structures of bulk gallium and tin, respectively [17]. Studies of superconductivity in the bulk Ga-Sn alloy [15] revealed a single, sharp, superconducting transition at low tin-concentration, whose temperature increased with an increasing tin amount. Then, further increasing the tin composition close to and above the eutectic point, resulted in the transition temperature remaining near 4.2 K, which is slightly above the superconducting temperature of pure bulk tin (3.72 K). In contrast to the results obtained for bulk Ga-Sn, we observed the onset of superconductivity for the nanoalloy at a temperature much higher than the critical temperature of bulk tin (inset in Figure 2a). Such a high temperature cannot be related to the formation of the Ga-rich segregated phase with a structure of α-Ga, as superconductivity in it emerges below 1.08 K [18]. The second step occurs at $T_{C2}$ = 4.7 K. This temperature is higher than the superconducting temperature for bulk tin and the maximal temperatures observed in the bulk Ga-Sn alloy [15]. Note that the nanostructured pure tin demonstrated superconducting temperatures up to 4.2 K [19] and 4.9 K [20]. Therefore, we can suggest that the transition at $T_{C2}$ = 4.7 K observed in the present study can be ascribed to the Sn-rich segregates, due to the combined impacts of size effects and alloying.

For pure gallium embedded into nanopores, many structures different from the stable α-Ga were reported [21,22,23], in agreement with the gallium capability to polymorphism. Besides the stabilized-within-nanopores β-Ga, other structures were found, which were not known for bulk gallium. Those structures demonstrated superconductivity at a higher temperature than α-Ga [14,21,24,25]. The X-ray pattern in Figure 7b gives information about the structure of the Ga-Sn alloy under the opal nanoconfinement. Most of the peaks belong to the tetragonal tin with a space group *I*4_1_/*amd*. These peaks are marked with asterisks. A peak at 2*θ* = 46.66° marked with a circle can be attributed to the disordered α-Ga, as in [21,22,23]. The peak at 2*θ* = 66.81°, marked with a diamond, was observed for gallium within nanopores in [14,22,23]. This peak was shown to belong to a tetragonal gallium structure [23], which was named λ-Ga in [21]. The other peaks of α-Ga and λ-Ga are not seen in Figure 7, likely due to the strong disorder of these structures. The superconducting temperature for λ-Ga is between 6.2 and 6.5 K, depending on the nanoporous matrices [14]. Therefore, we can suggest that the first superconducting transition shown in Figure 2 appears because of the emergence of λ-Ga. The low intensity of the peak agrees with weak diamagnetic screening below this transition. The peak at 57.99° was not reported for gallium earlier. It can be attributed to the emergence of a new structure, specifically for the Ga-Sn alloy within nanopores.

The magnetization behavior observed in the present study corresponds to the behavior of type-II superconductors. The strong difference between the ZFC and FCC curves below the second superconducting transition and the irreversible M-H hysteresis at 2 K evidence the strong pinning of vortices, as in dirty type-II superconductors. In contrast, the vortex pinning between the first and second transitions is much weaker, in accordance with the partly reversible hysteresis at 5 K and the weaker divergence of the ZFC and FCC curves. This can be understood if we assume that the first transition corresponds to the onset of superconductivity in a set of small, rather homogeneous segregates, with the λ-Ga structure.

We should emphasize the thermal hysteresis loops between the FCC and FCW curves shown in Figure 5. These loops are highly reproducible. The emergence of such a hysteresis is not a common phenomenon. Another kind of thermal hysteretic behavior caused by the paramagnetic Meissner effect or antiferromagnetic transition was observed for the FCC and FCW susceptibilities in bulk superconductors [26,27,28]. A thermal hysteresis similar to that shown in Figure 5 was considered theoretically for type-II superconductors in [29,30] on the base of Bean’s critical state model and vortex freezing upon cooling. The model was successfully applied to the polycrystalline and powder samples of Nb_3_Sn and YBa_2_Cu_3_O_7_ [31]. However, the model predicts merging the FCW and ZFC curves in the range of the hysteresis that does not agree completely with our results (Figure 5a).

The upper critical-field lines in type-II superconductors commonly have a negative curvature and can be modeled using, for instance, the two-fluid model [32]

(1) $$H_{C2}(T)=H_{C2}(0)\left(1-{\left(\frac{T}{T_{C0}}\right)}^{2}\right)$$

where $H_{C2}(T)$ and $H_{C2}(0)$ are upper critical-fields at temperatures *T* and 0, respectively, and $T_{C0}$ is a transition temperature at zero field. The critical line for the first superconducting transition fitted using Equation (1) for high magnetic fields is shown in Figure 4 by the dashed line. Its gives $H_{C2}(0)$ = 3.2 kOe. Then the coherence length can be estimated, based on the Ginzburg–Landau theory

(2) $$\xi (0)=\sqrt{\frac{\Phi_{0}}{2\pi H_{C2}(0)}}$$

where $\Phi_{0}$ is the flux quantum. Equation (2) gives the estimate ξ(0) = 18 nm. This value is of the order of the pore sizes in the opal template, which indicates that the coherence length is apparently limited by the electron free-path length. The upper critical-field and coherence length cannot be estimated reliably for the second superconducting transition because the temperature range of the negative curvature is narrow.

The critical lines within the temperature ranges of the positive curvature for both superconducting transitions allow fitting using the model [16], which takes into account the proximity effect. The results of fitting are shown in Figure 4, by solid lines. Note that the model [16] was used successfully to treat the positive curvature of the upper critical-field line for some other metals and alloys within nanopores [7,13].

## 5. Conclusions

Studies of dc magnetization in the binary Ga-Sn eutectic-alloy introduced into regular pores of the opal template revealed two superconducting transitions with temperatures of 6.4 and 4.7 K at 10 Oe, in contrast to the bulk alloy. We ascribed the first transition at 6.4 K to be in agreement with the X-ray pattern of the emergence of a small number of Ga-rich segregates with a structure of λ-Ga, which was reported for pure gallium within nanopores. The second transition, at 4.7 K, was ascribed to the Sn-rich segregates. It was suggested that the superconducting temperature for these segregates increased compared to the bulk alloy due to size effects. The H-T phase diagram demonstrated crossovers from the positive curvature of the upper critical-field lines at low fields to the common negative curvature at higher fields for both superconducting transitions. The positive curvature was treated using a model which considered the proximity effect. The uncommon thermal hysteresis was found between the FCC and FCW susceptibility curves for magnetic fields from 50 to 200 Oe. The hysteretic behavior generally agreed with the predictions of a model based on the critical state model and vortex kinetics.

## Data Availability

Not applicable.
